# *Tetraclita ehsani* sp. n. (Cirripedia, Tetraclitidae), a common intertidal barnacle from the Gulf of Oman, Iran

**DOI:** 10.3897/zookeys.136.1772

**Published:** 2011-10-13

**Authors:** Adnan Shahdadi, Benny K.K. Chan, Alireza Sari

**Affiliations:** 1 Department of Biology, Faculty of Science, University of Hormozgan, Bandarabbas, Iran; 2 Biodiversity Research Center, Academia Sinica, Taipei 115, Taiwan; 3 Zoological Museum, School of Biology, College of Science, University of Tehran, Tehran, Iran

**Keywords:** Crustacea, Cirripedia, Tetraclitidae, barnacles, Indian Ocean, Gulf of Oman, Iran

## Abstract

A new species of intertidal acorn barnacle *Tetraclita ehsani* **sp. n.** was identified from the Iranian coast in the Gulf of Oman. *Tetraclita ehsani* **sp. n.** inhabits low exposed rocky shores and also attaches to shells of molluscs and the barnacle *Megabalanus* species. Parietes of *Tetraclita ehsani* ranged from white to pink which is different from *Tetraclita serrata* (in South African waters), which has green parietes. Morphology of the tergum and cirrus III of *Tetraclita ehsani* **sp. n.** is distinctive from other described West Indian Ocean species which have pink or white parietes (*Tetraclita rufotincta*, *Tetraclita achituvi* and *Tetraclita reni*). The tergum of *Tetraclita ehsani* is very narrow and the basal margin is slightly concave or straight, in contrast to *Tetraclita rufotincta* and *Tetraclita reni*, in which the tergum are board and with a very concave basal margin. Cirrus I anterior ramus of both *Tetraclita ehsani* and *Tetraclita reni* is antenniform and thus differing from the cirrus I of *Tetraclita rufotincta* (see Chan et al. 2009). Cirrus III of *Tetraclita ehsani* **sp. n.** is non-antenniform and lacks multicuspidate type setae, which is different from *Tetraclita reni* by having an antenniform cirrus III and with multicuspidate setae.

## Introduction

*Tetraclita* species are common rocky intertidal acorn barnacles in the tropical and subtropical waters of the world (Newman and Ross 1976). *Tetraclita squamosa* Bruguière, 1789 had been recorded worldwide and was considered to be composed of nine sub-species due to high degree of morphological variations (Newman and Ross 1976). *Tetraclita squamosa* has since been split into 23 species using morphological and molecular approaches (Chan et al. 2007a, b, c), but the taxonomy of the species in the West Indian Ocean has still received scant attention (Chan et al. 2009). Pilsbry (1916) described *Tetraclita rufotincta* (= *Tetraclita squamosa rufotincta* Pilsbry, 1916) from Yemen and Zanzibar, and designated Yemen as the type locality. *Tetraclita rufotincta* was subsequently recorded in the northwest coast of India (Wagh 1971, 1972), the Red Sea (Achituv and Barnes 1978) and the Persian Gulf (Utinomi 1969). Ross (1999) additionally described *Tetraclita achituvi* Ross, 1999 and *Tetraclita barnesorum* Ross, 1999 from *Tetraclita rufotincta* in the Red Sea but subsequent molecular studies (Appelbaum et al. 2002) revealed *Tetraclita barnesorum* was a synonym to *Tetraclita rufotincta*. Ren (1989) described *Tetraclita africana* from Madagascar, but as this name was preoccupied and it was renamed *Tetraclita reni* Chan, Hsu & Tsai, 2009 by Chan et al. (2009). In the West Indian Ocean, *Tetraclita reni* is distributed in southern Madagascar and adjacent waters (Chan et al. 2009).

Taxonomic studies of Iranian barnacles after Utinomi (1969) are scant (Jones 1968; Southward and Newman 2003). Recently, extensive barnacle collections was carried out by Shahdadi (2007), Shahdadi and Sari (2011) on intertidal barnacles of the Persian Gulf and Gulf of Oman. *Tetraclita rufotincta* is common in the intertidal of the Persian Gulf (Shahdadi 2007). However, *Tetraclita* specimens collected from exposed rocky shores at the Gulf of Oman, Iran were morphologically different from other known *Tetraclita* species of the West Indian Ocean, suggesting that this is a new species. The *Tetraclita* specimens from the Gulf of Oman were examined by one of us (BKK Chan) using COI molecular markers, which showed a large genetic divergence from all known species in the West Indian Ocean (sequence of *Tetraclita* specimen from Gulf of Oman submitted to GenBank, unpublished data for phylogenetic comparisons). This further confirms the *Tetraclita* collected from the Gulf of Oman is a new species and described herein.

## Materials and methods

*Tetraclita* specimens were collected from the low intertidal shores at Ramin, Chabahar (25°16'N, 60°44'E) and Tis, Chabahar Bay (25°16'N, 60°40'E), Gulf of Oman, Iran. Barnacles were preserved in 95% Ethanol upon collection. The opercular plates, cirri and mouth parts were dissected and observed under compound light microscopes. The first three pairs of cirri and mouth parts were further investigated using a FEI Quanta 200 Scanning Electron Microscope (SEM) following Chan et al. (2007a, c, 2009). Terminology in describing the setae follows Chan et al. (2008). The COI barcode region was sequenced from the somatic body of the *Tetraclita* (paratype, ASIZCR 000231) collected from Chabahar, Gulf of Oman. DNA extraction and PCR protocol followed Chan et al. (2007a, c) and sequence was deposited in the GenBank.

The holotype and two paratypes are deposited in the Zoological Museum, University of Tehran (ZUTC) and one of the paratype was deposited in the Biodiversity Museum, Academia Sinica, Taiwan (ASIZCR).

## Systematics

### Superfamily Tetraclitoidea Gruvel, 1903. Family Tetraclitidae Gruvel, 1903. Tetraclitinae Gruvel, 1903. Tetraclita Schumacher, 1817

#### Tetraclita ehsani sp. n.

urn:lsid:zoobank.org:act:9829EE59-762C-4557-81AE-CB51DA038234

http://species-id.net/wiki/Tetraclita_ehsani

**Figure 1.**
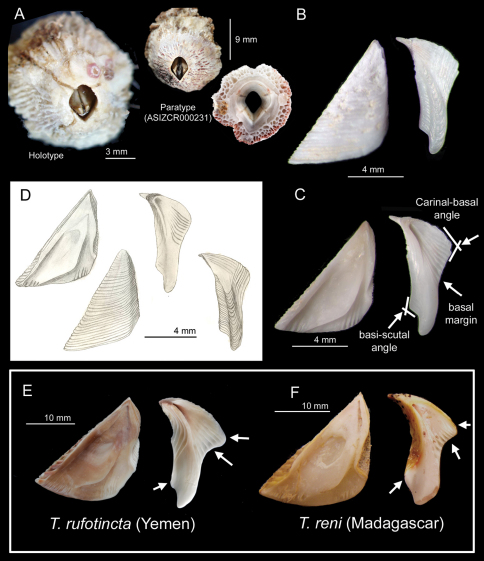
*Tetraclita ehsani* sp. n. **A** external parietes of the holotype and the top and basal view of the parietes of a paratype (ASIZCR 000231); note the basal view showing the parietal tubes **B** external view of scutum and tergum of the paratype (ASIZCR 000231) **C** internal view of scutum and tergum, (ASIZCR 000231); arrows indicate the diagnostic features of tergum from other Western Indian Ocean species (see table 1) **D** drawing of the holotype of the scutum and tergum **E** Scutum and tergum of *Tetraclita rufotincta* collected from Yemen (type locality) (after Chan et al. 2009) **F** scutum and tergum of *Tetraclita reni* collected from Madagascar (type locality) (after Chan et al. 2009).

Figures 1 [Fig F2] [Fig F3] [Fig F4] 5

##### Material examined.

HOLOTYPE. ZUTC-Cirri 1275, 1 specimen, Ramin, Chabahar, Gulf of Oman, Iran (25°16'N, 60°44'E), basal carino-rostro diameter 19.64 mm, height 13.74mm, orifice diameter 5.24mm. PARATYPE. ASIZCR 000231, 1 specimen, locality same as holotype, basal carino-rostro diameter 18.16 mm, height 11.91 mm, orifice 5.78 mm. PARATYPE. ZUTC-Cirri. 1276, 1 specimens, locality same as holotype. from Ramin (type locality). PARATYPE. ZUTC-Cirri. 1277, 1 specimen, Tis (Portuguese Castle), Chabahar Bay, Gulf of Oman, Iran (25°16'N, 60°40'E). GenBank accession number of paratype ASIZCR 000231: JN603678.

##### Diagnosis.

 Parietes white or pink, tergum very narrow, basal margin slightly concave or almost straight, tergal spur long and narrow. Mandible with five teeth, labrum with 4 large sharp teeth on each side of the cutting edge. Anterior ramus of cirrus I antenniform.

##### Description.

*Parietes* conical, white to pink or white with pink ribs (Fig. 1A), radii and alae narrow, sheathstriate, parallel to base and about ½ height of wall, sheath white to dirty white, or pink. Parietes composed of 3-4 rows of honey comb parietal tubes (Fig. 1B). *Scutum* and *tergum* white (Fig. 1B, C, D). *Scutum* narrow, 1.5 times higher than wide, lower half of occludent margin with >10 oblique teeth, articular ridge sinuous, adductor ridge extremely developed, angular and extending to basal margin, adductor muscle pit shallow, seven distinct rostral and four to six lateral depressor crests (Fig. 1B, C, D), external surface smooth with faint horizontal striations (Fig. 1). *Tergum* long and narrow (length more than twice as width) with ten definite depressor crests, scutal margin slightly concave, spur long and narrow, external spur surface with a medial furrow, basi-scutal angle sharp and about 117.8°, upper carinal margin convex and basal margin slightly concave or straight (Fig. 1B, C, D). Carinal-basal angle (angle between the carinal and basal margin) is ~103° (Fig. 1C).

**Figure 2. F2:**
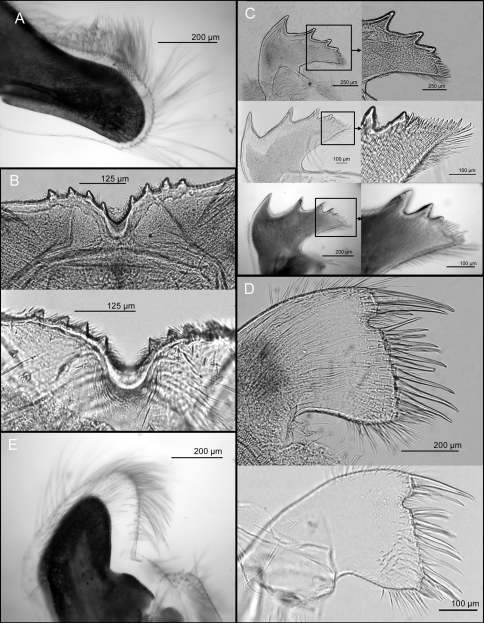
*Tetraclita ehsani* sp. n. Light microscopy showing: **A** mandibular palp **B** labrum (two individuals) **C** mandibles (3 individuals) and with enlarged views of lower margin and inferior angle **D** maxillule (2 individuals) and **E** maxilla.

**Figure 3. F3:**
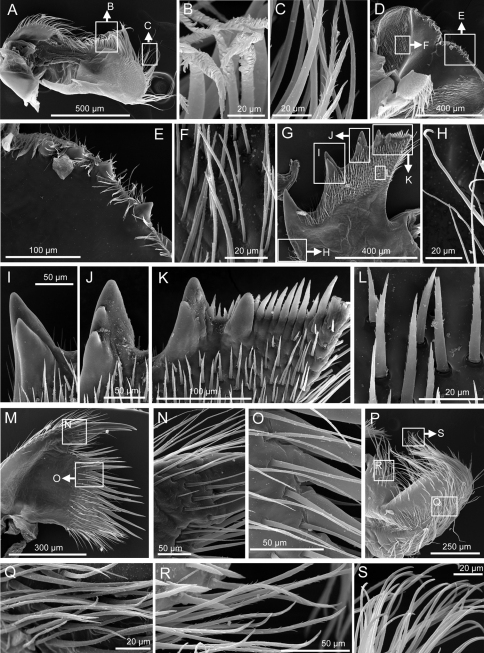
*Tetraclita ehsani* sp. n. SEM showing: **A** mandibular palp **B,** **C** serrulate type setae on mandibular palp **D** labrum **E** cutting edge of labrum showing the 4 large sharp teeth **F** simple type setae on inner side of labrum **G** mandible **H** simple setae on the lateral side of the mandible **I** second bi-dentated tooth of the mandible **J** third tri-dentated tooth of the mandible **K**, fourth and fifth tooth of the mandible **L**, serrulate blade shaped setae on mandible **M** Maxillule **N, O** serrulate setae on maxillule surface **P** maxilla **Q, R**, serrulate setae on maxilla **S** simple setae on tip of maxilla.

*Mandibular palps* elongate, setae on superior margin only, simple type setae at tip and serrulate setae at the middle region of the superior margin (Figs 2A, 3A, B, C). *Labrum* notched, notch shallow, four erect large teeth on each side of the cutting edge (Figs 2B, 3E–H). *Mandible* with five teeth excluding the inferior angle, first tooth separated from the remaining teeth, second and fourth teeth bidentate, third teeth tridentated fifth tooth small and located close to the fourth tooth, lower margin with >10 setae, height of setae similar to height of the fifth tooth, inferior angle sharp, with two large setae on tip, mandible surface with blade shaped serrulate type setae (Figs 2C, 3G–L). *Maxillule* notched, with two large and four small simple setae above notch, 11 setae in median cluster and 10 small and slender simple setae on the cutting margin below notch (Figs 2D, 3M–O). *Maxilla* bi-lobed, serrulate type setae at both lobes (Figs 2E, 3P–S).

**Figure 4. F4:**
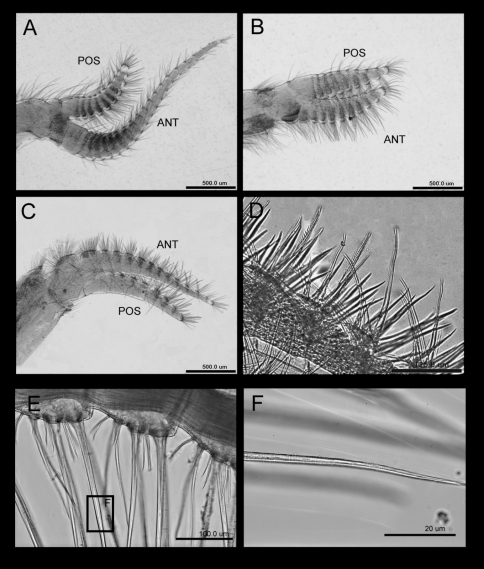
*Tetraclita ehsani* sp. n. Light microscopy showing: **A** cirrus I **B** cirrus II **C** cirrus III **D** bidentate serrulate type setae on rami of cirrus III **E** intermediate segment of anterior ramus of cirrus VI **F** Long serrulate setae at cirrus VI. ANT – Anterior ramus, POS – Posterior ramus.

**Figure 5.**
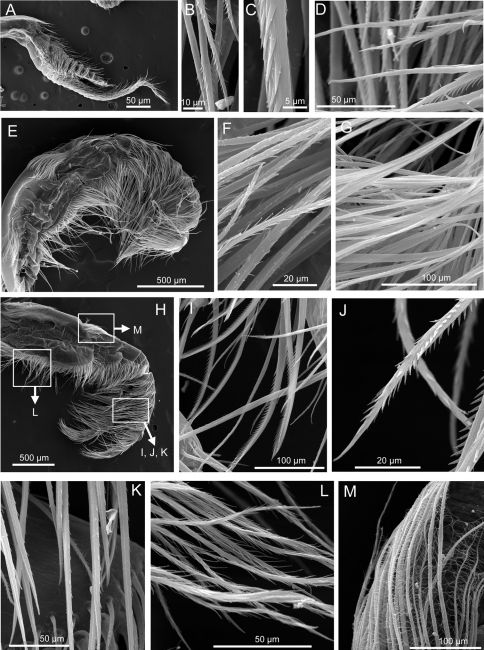
*Tetraclita ehsani* sp. n. SEM showing: **A** cirrus I **B, C, D**, serrulate type setae on rami of cirrus I **E** cirrus II **F, G** serrulate type setae on rami of cirrus II **H** cirrus III **I, J, K**, serrulate type setae on rami of cirrus III **L** serrulate type setae on protopod of cirrus III **M** pappose setae on protopod of cirrus III.

*Cirrus I* anterior ramus antenniform, twice as long (27 segments) as posterior ramus (10 segments) (Figs 4, 5A), both rami with a feathery serrulate type setae (3–4 rows dense setules in each seta) and a serrulate type setae (very sparse setule along the seta) (Fig. 5B–D). *Cirrus II* with shorter rami, anterior and posterior ramus similar in length (each with 10 segments), with serrate and simple setae (Fig. 5E–G). *Cirrus III* with longer anterior ramus (anterior and posterior ramus 13 and nine segments respectively; Fig. 4). Both rami bear bidentate serrate setae, feathery serrulate setae and blade shaped serrulate setae, protopod bears pappose setae with long feather seta (Fig. 5H–M). *Cirrus IV–VI* ctenopods, cirrus IV with 17 segments for both anterior and posterior rami, cirrus V, anterior ramus 21 segments, posterior ramus 19 segments, cirrus VI, anterior ramus 18 segments, posterior ramus 22 segments. Intermediate segment of anterior ramus of cirrus VI bears 3 pairs of long serrulate setae and 3 pairs of short setae (Fig. 4E, F).

##### Etymology.

This species is named in honour of Ehsan Entezari-Zarch, B.Sc. student in Animal Biology at the University of Tehran, who unfortunately passed away during a field collection in October 2009.

##### Habitat.

 This species was present at the exposed low shores at intertidal zone, attaching on rocks but sometimes were observed on mollusk shells and on the shell surface of the barnacle *Megabalanus* species at the Gulf of Oman.

##### Distribution.

 At present, only known from the Iranian coast in the Gulf of Oman and absent from the Persian Gulf (see Shahdadi 2007).

## Discussion

*Tetraclita ehsani* sp. n., from Iranian waters, shows diagnostic morphological characters that distinguish it from other known species in the Western Indian Ocean (*Tetraclita rufotincta*, *Tetraclita reni*, *Tetraclita achituvi* and *Tetraclita serrata*). All the *Tetraclita* in the West Indian Ocean have white to pink parietes except *Tetraclita serrata* Darwin, 1854 which has green parietes. In addition to the colour of the parietes, *Tetraclita serrata* has serrated lines on parietes surface and with a broader spur in tergum, when compared to *Tetraclita ehsani*. It is difficult to distinguish *Tetraclita ehsani* from *Tetraclita reni*, *Tetraclita achituvi* and *Tetraclita rufotincta* using the external shell morphology. *Tetraclita ehsani* can be, however, distinguished from the other species by the tergum morphology and arthropodal characters. The tergum of *Tetraclita ehsani* is very narrow and the basal region is slightly concave or almost straight, contrasting to the tergum of *Tetraclita rufotincta* and *Tetraclita reni*, which are board and with a strongly concave basal margin (Fig. 1E, F). The basi-carinal angle of *Tetraclita ehsani* sp. n. is ~100°, which is larger than that in *Tetraclita reni* (80°) and *Tetraclita rufotincta* (73°; Fig. 1C, D, E). The basi-scutal angle of the tergum of *Tetraclita ehsani* is ~120°, more angular than that of *Tetraclita reni* (150°) (Fig. 1C, D, E; see Chan et al. 2009). Anterior ramus of the cirrus I of both *Tetraclita ehsani* and *Tetraclita reni* is antenniform, thus differing from *Tetraclita rufotincta* (see Chan et al. 2009). Cirrus III *Tetraclita ehsani* sp. n. is non-antenniform and lacks multicuspidate setae, which is different from *Tetraclita reni*, in which the both anterior and posterior rami are antenniform and possess multicuspidate setae (see Chan et al. 2008) (Table 1).

**Table 1.** Morphological comparison of *Tetraclita ehsani* sp. n. with other *Tetraclita* from the west Indian Ocean. *Tetraclita serrata* was not included into comparison as the shell colour of *Tetraclita serrata* is green, which is obviously different from other West Indian Ocean species. For morphology features of *Tetraclita rufotincta*, *Tetraclita reni* and *Tetraclita achituvi*, see Pilsbry (1916), Chan et al. (2009) and Ross (1999).

**Characters**	***Tetraclita ehsani* sp. n.**	***Tetraclita rufotincta* Pilsbry, 1916**	***Tetraclita reni* Chan et al., 2009**	***Tetraclita achituvi* Ross, 1999**
Shell colour	White to pink	Pink to grey	White to pink	Pink to white
Tergum colour and length	White, twice longer than wide	Yellow to pink, longer than wide (less than twice)	Yellow to pink, longer than wide (less than twice)	White,longer than wide(about twice)
Tergum Spur	Longer than wide(~1.2 times)	Wider than long (~1.2 times)	Wider than long (about twice)	Wider than long (1.5 times)
Basal margin of tergum	Slightly concave with no clear angle	Strongly concave, forming almost right angle	Strongly concave, forming almost right angle	Slightly concave
Adductor muscle pit in scutum	shallow	deep	shallow	deep
Adductor ridge in scutum	Extremely developed, angular and extending to basal margin	Developed but not extending to basal margin	Developed but not extending to basal margin	Extremely developed
Labrum teeth	Four, large, sharp and erect on each side of notch	Absent or four small blunt on cutting margin	Four small blunt teeth at each side of the cutting margin	Six small teeth in notch
Cirrus III	Non-antenniform, without multicuspidate setae	Non-antenniform, without multicuspidate setae	Antenniform, with multicuspidate setae	Antenniform, without multicuspidate setae

The biogeography of *Tetraclita* species in the West Indian Ocean appears to be distinctive between different oceanographic systems. *Tetraclita rufotincta* has the widest distribution, covering the Persian Gulf, the Red Sea and the East African coast and absent from South Africa and southern Madagascar. *Tetraclita reni* is confined to southern Madagascar and adjacent waters and *Tetraclita achituvi* has been reported only from the Red Sea. *Tetraclita ehsani* has not been recorded in other parts of the Western Indian Ocean, except from the Iranian coast in the Gulf of Oman and it is absent from the Persian Gulf. It may be possible that *Tetraclita ehsani* is common in the Arabian Sea. It is essential to conduct further biodiversity surveys in the Arabian Sea region, including the west coast of India (see Wagh 1972) to ascertain the geographic distribution of *Tetraclita ehsani*.

## Supplementary Material

XML Treatment for Tetraclita ehsani
